# Hyaluronan-Binding Protein Promotes Fibroblast Transformation and Heart Failure by Modulating the STAT5A–MMP13 Pathway

**DOI:** 10.3390/biomedicines13061302

**Published:** 2025-05-26

**Authors:** Hui Yan, Bing Huang, Bofang Zhang, Yunyao Li, Qiping Zhou, Ayipali Abudoureyimu, Guiqiu Cao, Hong Jiang

**Affiliations:** 1 Department of Cardiology, The Fifth Affiliated Hospital, Xinjiang Medical University, Urumqi 830000, China; 2 Hubei Key Laboratory of Cardiology, Wuhan 430000, China; 3 Cardiovascular Research Institute, Wuhan University, Wuhan 430000, China; 4 Department of Cardiology, Renmin Hospital, Wuhan University, Wuhan 430000, China

**Keywords:** myocardial infarction, heart failure, cardiac remodeling, hyaluronan-binding protein involved in hyaluronan, cardiac fibrosis

## Abstract

**Background:** Adverse cardiac remodeling drives heart failure progression, but the role of hyaluronan-binding protein (HYBID) in this process remains unclear. This study investigated the role of HYBID as a key profibrotic factor in the progression of adverse cardiac remodeling with a focus on its functional impact on cardiac fibroblasts and underlying molecular mechanisms. **Methods:** RNA sequencing analysis was employed to identify differentially expressed genes in mouse ventricular tissue post-myocardial infarction (MI). Fibroblast-specific genetically modified mouse models (knockdown and overexpression) were generated using FSP1 promoter-driven adeno-associated viruses. Comprehensive histological and biochemical assessments were conducted both in vivo and in vitro to evaluate the effects of HYBID modulation on cardiac remodeling. Molecular docking and immunoprecipitation assays were utilized to elucidate the mechanistic interactions between HYBID and its downstream targets. **Results:** RNA sequencing revealed HYBID as a fibroblast-enriched protein significantly upregulated in myocardial tissue of MI mice. Fibroblast-specific knockdown of HYBID attenuated MI-induced fibroblast activation, improved cardiac function, and mitigated adverse cardiac remodeling. Conversely, HYBID overexpression exacerbated fibroblast activation and promoted cardiac remodeling. Mechanistically, HYBID was found to competitively bind to STAT5A, thereby inhibiting the anti-fibrotic effects of MMP13 and driving fibroblast activation and adverse remodeling post-MI. **Conclusions:** Our findings establish HYBID as a novel fibroblast-enriched regulator that exacerbates fibrosis and adverse cardiac remodeling following MI. By uncovering the HYBID–STAT5A–MMP13 axis as a critical signaling pathway, this study provides new insights into the molecular mechanisms underlying heart failure progression.

## 1. Introduction

Adverse cardiac remodeling represents a critical pathological process that underlies the progression to ischemic heart failure and poor clinical outcomes. Despite significant advancements in the management of myocardial infarction (MI), the structural and functional deterioration of the heart remains a major challenge, contributing to high morbidity and mortality worldwide [1,2]. The extent of infarction and the adequacy of the post-MI reparative response are fundamental determinants of cardiac remodeling, a process characterized by complex interactions among cellular and molecular pathways [1,2]. Central to this process is cardiac fibrosis, which reflects the activation of both reparative and maladaptive mechanisms. Activated cardiac fibroblasts (CFs) and their differentiated counterparts, myofibroblasts, play pivotal roles in fibrosis by driving excessive extracellular matrix (ECM) deposition [2,3,4,5]. This pathological ECM accumulation leads to interstitial expansion, reduced tissue compliance, and ultimately, end-stage heart failure [2,3,4,5]. Consequently, targeting the persistent activation of CFs has emerged as a promising therapeutic strategy to mitigate adverse cardiac remodeling post-MI. However, the key molecular triggers initiating CF differentiation and the precise mechanisms governing cardiac remodeling remain incompletely understood, underscoring the need for further investigation.

Hyaluronan-binding protein involved in hyaluronan (HYBID) was initially identified as an inner ear-specific protein [6]. As a secreted protein localized to the cytoplasm, HYBID binds to hyaluronan and facilitates its depolymerization, thereby playing a critical role in ECM remodeling [7,8]. Beyond its initial discovery, HYBID has been implicated in promoting the proliferation, invasion, and adhesion of various tumor cell lines, highlighting its broader functional significance [9,10,11]. Emerging evidence further suggests that HYBID is a key player in the pathogenesis of fibrotic diseases, including cartilage fibrosis [12,13] and pulmonary fibrosis [14]. Downregulation of HYBID has been shown to reduce collagen production and suppress cell proliferation and migration, suggesting its potential as a therapeutic target in fibrosis [14]. Although preliminary studies have hinted at a role for HYBID in myocardial fibrosis [15], the precise regulatory mechanisms underlying its involvement in cardiac remodeling remain elusive.

Matrix metalloproteinases (MMPs), a family of zinc-dependent endopeptidases, are essential regulators of ECM homeostasis and tissue repair [16]. Among these, MMP13 (collagenase 3) is particularly noteworthy for its ability to degrade fibrillar collagens (types I, II, and III), fibronectin, and aggrecan, thereby counteracting fibrosis [17,18]. Studies in ApoE-deficient murine models have demonstrated that MMP13 knockout exacerbates collagen accumulation in atherosclerotic lesions, underscoring its anti-fibrotic role [19,20]. Intriguingly, HYBID has been reported to exacerbate fibrosis in osteosarcoma by downregulating MMP13 [21], raising the possibility of a similar interaction in cardiac fibrosis. However, the relationship between HYBID and MMP13 in the context of myocardial remodeling has yet to be fully elucidated, leaving a critical gap in our understanding of the molecular pathways driving post-MI cardiac fibrosis.

In this study, we employed a discovery-driven, unbiased approach to identify HYBID as a key regulator enriched in CFs and upregulated during MI-induced cardiac remodeling. Using adeno-associated virus-mediated gene delivery, we demonstrated that HYBID knockdown significantly attenuated MI-induced cardiac dysfunction and adverse remodeling, whereas HYBID overexpression exacerbated these pathological changes. In vitro experiments further revealed that HYBID silencing effectively suppressed hTGF-β1-induced CF overactivation, while HYBID overexpression aggravated these effects. Through transcriptomic and functional analyses, we identified MMP13 as a novel downstream target of HYBID. Notably, the anti-fibrotic effects of HYBID knockdown were abrogated by MMP13 inhibition, suggesting a functional interplay between these molecules. Mechanistically, immunoprecipitation and molecular docking studies revealed that HYBID negatively regulates MMP13 expression by competitively binding to STAT5A. Collectively, these findings position HYBID as a potential therapeutic target for preventing myocardial fibrosis and adverse cardiac remodeling, offering new insights into the molecular mechanisms underlying heart failure progression.

## 2. Methods and Materials

### 2.1. Animal Experiments

Age-matched male C57BL/6 mice at 6–8 weeks of age were provided by the Animal Experiment Center of Renmin Hospital of Wuhan University (Wuhan, China). MI was experimentally induced via ligation of the left anterior descending (LAD) coronary artery, according to previously described methods [22,23]. Briefly, the mice were initially anesthetized with 2% isoflurane and then maintained under anesthesia with 1% isoflurane once the loss of the toe-pinch reflex was observed. A vertical incision was subsequently made in the cervical region to expose the trachea, facilitating tracheal intubation. After the skin in the left anterior chest was incised, the pectoralis major muscle was meticulously separated to access the third intercostal space. Finally, the LAD was ligated via a 6–0 silk suture. The sham-operated mice underwent the same surgical procedures except for LAD ligation. Following the surgical procedure, the mice were fed for 4 weeks. All the mice were housed at a temperature of 22 ± 2 °C with a standard 12 h dark/light cycle, and were provided ad libitum access to food and water.

All experimental animal procedures in this study were conducted in accordance with institutional ethical guidelines and were approved by the Animal Experiment Ethics Committee of Renmin Hospital of Wuhan University (Protocol No. WDRM 202 240805B).

### 2.2. Adeno-Associated Virus Construction and Injection

The adeno-associated virus (AAV) serotype 9, driven by the fibroblast-specific protein 1 (FSP1) promoter, was utilized to express the HYBID and STAT5A genes. The following constructs were generated: AAV-HYBID, AAV-shHYBID, and AAV-STAT5A. For comparative analysis, an AAV empty vector (AAV-NC or AAV-shNC) was used as a negative control (GeneChem, Shanghai, China). Fourteen days before the simulation of MI surgery, mice were administered AAV9 or empty vector via tail vein injection at a controlled slow rate, with a dosage of 1 × 10^12^ vg/mL and a volume of 200 μL per mouse.

### 2.3. Cardiac Fibroblast Isolation and Culture

CFs were isolated from neonatal mice as previously described [23,24]. In brief, 1–3-day-old neonatal C57BL/6 mice were sacrificed, and their hearts were harvested immediately. After being washed 2–3 times with D-Hank’s buffer, the hearts of lactating mice were cut into pieces of approximately 1 mm and transferred to 0.125% trypsin (G4010, Servicebio, Wuhan, China) at 4 °C overnight. The next day, predigested heart fragments were treated with 0.8% type II collagenase (1904MG100, BioFroxx, Einhausen, Germany) for an additional 17 min at 37 °C under constant shaking. The digested cell suspension was subsequently gently triturated by pipetting and then passed through a 20 μm cell strainer to eliminate tissue debris. Finally, all the digested cells were centrifuged, resuspended in DMEM/F12 (SH30126.01, Cytiva, Marlborough, MA, USA), supplemented with 10% fetal bovine serum (FBS, 10099, Gibco, Melbourne, Australia) and 1% penicillin-streptomycin (G4003, Servicebio, Wuhan, China), and then seeded into 10 cm plates. After incubation at 37 °C with 5% CO_2_ for 1.5 h, the supernatant was removed, and the cells that adhered to the bottom of the plates were considered CFs.

### 2.4. Transfection and Infection

CFs were seeded into six-well plates and allowed to achieve a 50% cell density. The cells were treated according to the corresponding transfection protocol.

Adenovirus: To achieve targeted knockdown or overexpression of specific genes, recombinant Ad-HYBID vectors were generated by introducing mouse HYBID cDNA into a replication-defective adenoviral vector. Concurrently, an adenoviral construct expressing a mouse-specific short hairpin RNA (shRNA) designed to downregulate HYBID was developed, referred to as Ad-shHYBID. Ad-NC and Ad-shNC were used as controls, and the efficiency of these adenoviruses was validated. CFs were transduced with Ad-shHYBID, Ad-shNC, or Ad-NC at a multiplicity of infection (MOI) of 20 for 24 h, whereas Ad-HYBID was used to transduce CFs at an MOI of 40.

Plasmid or siRNA: Expression plasmids encoding the full-length HA-tagged STAT5A and its truncated mutants were constructed using PCR and then cloned and inserted into the pcDNA3.1-HA vector, with the pcDNA 3.1-HA plasmid serving as a control. Small interfering RNA (siRNA) oligonucleotides against STAT5A (siSTAT5A) and negative control siRNA (siNC) were synthesized by GenePharma (Shanghai, China).

Following the manufacturer’s protocol, CFs were transfected with Lipo8000™ transfection reagent (Beyotime, Shanghai, China) to overexpress or silence STAT5A in vitro.

### 2.5. Cardiac Fibroblast Treatment

Myofibroblast differentiation was induced via the use of 10 ng/mL recombinant human transforming growth factor-β1 (hTGF-β1, HY-P7118, MCE, Shanghai, China), with an equal volume of phosphate-buffered saline (PBS) serving as a control. The culture medium was replaced with serum-free medium before treatment with 10 ng/mL hTGF-β1, followed by incubation at 37 °C and 5% CO_2_ for 24 h.

### 2.6. Quantitative Real-Time PCR (qPCR)

Total RNA from cells or cardiac tissue was extracted via a SteadyPure Universal RNA Extraction Kit (AG21017, Agbio, Beijing, China). The RNA was subsequently reverse-transcribed into cDNA with SweScript All-in-One-First-Strand RT SuperMix (G3337, Servicebio, Wuhan, China). Then, qPCR was conducted with 2× Universal Bule SYBR Green qPCR Master Mix (G3326, Servicebio, Wuhan, China) on Real-Time PCR System (Applied Biosystems ViiA 7 Dx, Waltham, MA, USA). GAPDH was used as an internal reference. The sequences of primers used are listed in Supplementary Table S1.

### 2.7. Western Blot

Proteins were extracted from tissues or cultured cells as described previously [25]. Protein concentrations were determined using the BCA. Equal amounts of protein extracts were subsequently separated on 10% SDS–polyacrylamide gels via electrophoresis and then transferred to poly vinylidene fluoride (PVDF) membranes. Then, the membranes were blocked with 5% skim milk in Tris-buffered saline with Tween 20 for 2 h at room temperature. Afterwards, the protein samples were individually subjected to incubation with their respective primary antibodies overnight at 4 °C (Supplementary Table S2). On the following day, after washes with TBST, the membranes were exposed to horseradish peroxidase (HRP)-conjugated anti-rabbit or anti-mouse secondary antibodies for 1.5 h at room temperature. Finally, the protein signals of the members were visualized by enhanced chemiluminescence (BL523, Biosharp, Hefei, China), and images were obtained with a ChemiDoc™ Imaging System (Bio-Rad, Hercules, CA, USA). The density of the protein bands was quantified via ImageJ 1.0 after normalization to that of GAPDH.

### 2.8. Cellular Functions

Cell proliferation assay: Cell proliferation was assessed using the CCK8 assay (C0038, Biyuntian, Nanjing, China) following the manufacturer’s protocol. The cells were plated in 96-well plates at a density of 5000 cells per well and allowed to adhere and proliferate in complete growth medium overnight. After 24 h incubation, 10 µL of the Cell Counting Kit-8 reagent was added to each well. The plates were subsequently returned to the incubator and maintained at 37 °C for 1 to 2 h to facilitate the colorimetric reaction, which serves as an indicator of cell viability and proliferation. The absorbance (OD value) was measured at 450 nm via a Tecan Infinite 200Pro (Tecan, Männedorf, Austria) at several time points post treatment: 0 h, 12 h, 24 h, and 48 h.

Scratch Migration Assay: The migratory capacity of CFs was evaluated by scratch assay following HYBID modulation. CFs were cultured in 6-well plates. When 70–80% confluence was reached, a straight scrape was made in the middle of each well via a pipette tip. Next, the medium was replaced with serum-free medium. Images of the scraped areas were captured at two time points: immediately after scraping (0 h) and after a 24 h incubation at 37 °C. Cell migration ability was evaluated on the basis of the migration distance (difference in scratch width before and after migration), which was measured using ImageJ 2.0 (National Institutes of Health, Bethesda, MD, USA).

### 2.9. Echocardiography

The cardiac function and structure of the mice were assessed using echocardiography (Vinno Technology, Shenzhen, China) with a 23 MHz linear transducer, both preoperatively and at 4 weeks post-surgery. The mice were anesthetized via isoflurane (1–2%) and placed on a 37 °C heating pad in the supine position. These echocardiography parameters such as the left ventricular ejection fraction (LVEF) and fractional shorting (LVFS) were collected from three to five cardiac cycles.

### 2.10. Immunofluorescence Staining

Immunofluorescence staining was performed on CFs grown on coverslips. The cells were fixed in 4% paraformaldehyde for 15 min and blocked with 3% BSA in PBS for 30 min at room temperature. Next, the cells were incubated with primary antibody or PBS (used as a negative control) overnight at 4 °C (Supplementary Table S2). The appropriate Alexa Fluor-conjugated secondary antibody was subsequently used for fluorescence staining. Cell nuclei were stained with DAPI (1 μg/mL, GB1012, Servicebio, Wuhan, China) for 10 min at room temperature. Confocal microscopy images were acquired using a Zeiss confocal laser scanning microscope (NIKON Eclipse TI, Tokyo, Japan). Additionally, other fluorescent immunocytochemical images were captured using a standard fluorescence microscope (IX71, Tokyo, Japan).

### 2.11. Coimmunoprecipitation

Coimmunoprecipitation (COIP) was performed as previously described with minor modifications [26]. Briefly, HEK293-T cells were lysed in lysis buffer with PMSF and cocktail on ice for 30 min. Next, clear lysates were incubated with immunoprecipitation (IP)-grade antibodies for a period of 1 h. The target proteins were subsequently precipitated via magnetic protein A/G beads (catalogue number L-1004, Biolinkedin, Shanghai, China) in conjunction with the appropriate antibodies. Finally, Western blot was performed to detect the immune complexes using the designated primary and secondary antibodies.

### 2.12. Histological Staining

Prior to tissue collection for histological analysis, the mice were sacrificed under CO_2_ anesthesia. Body weight (BW), heart weight (HW), and lung weight (LW) were measured to assess the HW/BW and LW/BW ratios. Subsequently, the isolated heart tissues were immediately fixed in a 4% paraformaldehyde solution, followed by routine dehydration, paraffin embedding, and serial sectioning (4 μm). Masson’s trichrome staining and Sirius red staining were performed following established protocols. Sirius red and Masson’s trichrome staining were employed for the quantitative analysis of fibrotic and infarcted areas.

### 2.13. Collagen Gel Contraction Assays

The collagen gel contraction assay was performed using CFs. Cells were mixed with a neutralized solution of type I collagen (3.34 mg/mL) derived from rat tail (354236, Corning, NY, USA) to prepare collagen lattices. First, 0.2% acetic acid solution was prepared using 100% glacial acetic acid, followed by sterile filtration through a 0.2 μm filter, and the solution was cooled to 4 °C. Under sterile conditions, type I collagen was mixed with the 0.2% acetic acid solution to prepare a collagen solution at a concentration of 3 mg/mL, which was thoroughly mixed at 4 °C. To determine the NaOH titration conditions for collagen, the optimal amount of NaOH (8 μL) to be added to the collagen/medium mixture was experimentally determined, and the same amount of NaOH was used for all subsequent gel preparations. In the contraction assay, the final collagen concentration was 3.0 mg/mL, and the cell concentration was 1 × 10^5^ cells/mL. Each experiment was independently performed at least three times, and all conditions were repeated in triplicate to ensure reproducibility of the results.

### 2.14. Statistical Analyses

The data are expressed as the mean ± standard deviation (SD) for all the measured values. Normality was assessed using Shapiro–Wilk tests. For comparing two distinct groups, either the Student’s *t*-test or the Mann–Whitney U test was applied. Multiple group comparisons were performed by a one-way or two-way analysis of variance followed by Tukey’s multiple comparisons test. All the statistical analyses were conducted using SPSS 26.0 software, and graphical representations of the data were generated with GraphPad Prism 8.0 software.

## 3. Results

### 3.1. HYBID Is Upregulated in Mouse Models of Cardiac Fibrosis

**Figure 1:**
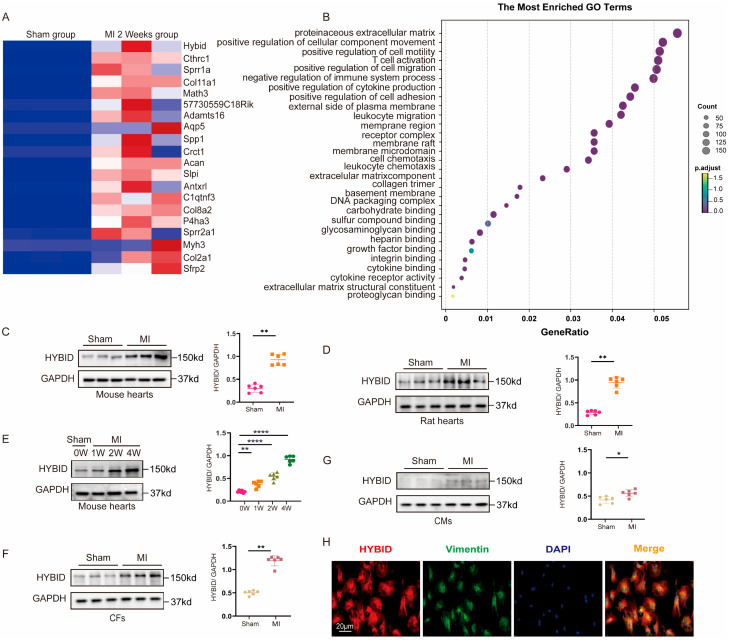
HYBID expression is upregulated in a mouse model of myocardial infarction (MI). (**A**) Heatmap of the top 20 upregulated genes in MI samples from RNA-seq analysis (log2(Fold-change) > 2, adjusted *p*-value < 0.05). (**B**) GO term enrichment analysis of upregulated in both sham and MI samples (log2(Fold-change) > 2, adjust *p*-value < 0.05). (**C**) Western blot and quantification of HYBID protein levels in both MI and sham samples (n = 6). (**D**) The rat hearts with or without MI were harvested for Western blot (n = 6). (**E**) Proteins of HYBID were elevated at 1 week, 2 weeks, and 4 weeks of MI mice (n = 6). (**F**) Western blot and quantification of HYBID protein levels in isolated CMs at 4 weeks after MI or sham (n = 6). (**G**) Western blot and quantification of HYBID protein levels in isolated CFs at 4 weeks after MI or sham (n = 6). (**H**) Immunofluorescence staining for co-localization of HYBID and vimentin (n = 6), Scale bar: 20 μm. * *p* < 0.05, ** *p* < 0.01, and **** *p* < 0.0001. (**C**,**D**,**F**,**G)** by Mann–Whitney U test; and (**E**) by two-way ANOVA followed by Tukey’s multiple comparisons test. (**A**,**B**) Adjusted *p*-values (Benjamini–Hochberg corrected; FDR < 0.05).

To systematically identify molecular targets and elucidate the mechanisms driving cardiac fibrosis following MI, we performed unbiased RNA sequencing (RNA-seq) analysis of cardiac tissues collected from MI-induced and sham-operated mice at 2 weeks post-surgery. Gene Ontology (GO) analysis (false discovery rate q-value < 0.01) revealed that differentially expressed genes were significantly enriched in biological processes related to ECM organization, cell migration, and other cardiac remodeling pathways (Figure 1B). These findings align with the known pathological features of cardiac fibrosis, suggesting that the transcriptomic changes observed are closely associated with post-MI tissue remodeling.

Subsequent analysis of the top 20 upregulated genes in the MI model identified HYBID as the most significantly altered gene, exhibiting a marked increase in expression compared to sham controls (Figure 1A). To validate the RNA-seq results, we independently assessed HYBID expression levels in cardiac tissues from MI and sham-operated mice. Consistent with the transcriptomic data, both mRNA and protein levels of HYBID were significantly elevated in the myocardium two weeks post-MI (Figure 1A,C). Furthermore, the upregulation of HYBID was not limited to mice; a similar increase in expression was observed in rat models of MI (Figure 1D), underscoring the potential conservation of this response across species. Intriguingly, the upregulation of HYBID expression became even more pronounced at four weeks post-MI (Figure 1E), suggesting a time-dependent role for HYBID in the progression of cardiac remodeling.

Based on these findings and supported by previous reports [27], we utilized a mouse model four weeks post-MI to further investigate the functional role of HYBID in cardiac fibrosis and its underlying mechanisms. The temporal increase in HYBID expression, coupled with its association with ECM organization and cell migration, strongly implicates HYBID as a key regulator of pathological cardiac remodeling following MI.

Cardiac remodeling following MI constitutes a complex pathological process, wherein cardiomyocytes (CMs) and CFs emerge as pivotal cellular entities driving this progression. To identify the specific cell populations responsible for the elevated expression of HYBID during cardiac remodeling, we isolated CMs and CFs from the hearts of adult mice subjected to MI and assessed HYBID expression using qPCR and Western blot analyses. Our results revealed a significant increase in both mRNA and protein levels of HYBID within CFs following MI (Figure 1F,G and Figure S1B). Although HYBID expression was modestly elevated in CMs post-MI, the most striking upregulation occurred in CFs, establishing CFs as the primary cellular source of HYBID during cardiac remodeling.

Consistent with these observations, immunofluorescence analysis demonstrated a pronounced upregulation of HYBID expression specifically in CFs of MI-induced mice relative to sham-operated controls. Confocal imaging revealed that HYBID was predominantly localized in the cytoplasm of vimentin-labeled CFs (Figure 1H). This subcellular distribution aligns with the potential functional role of HYBID in modulating intracellular signaling pathways or ECM interactions, both of which are critical to cardiac fibrosis and remodeling.

Collectively, these results highlight CF-derived HYBID as a key contributor to cardiac remodeling post-MI. The cell-specific upregulation and cytoplasmic localization of HYBID in CFs suggest its involvement in fibroblast activation and ECM remodeling, processes central to the pathogenesis of cardiac fibrosis. These findings provide a foundation for further investigation into the mechanistic role of HYBID in CFs and its potential as a therapeutic target for mitigating adverse cardiac remodeling.

### 3.2. HYBID Knockdown Ameliorates MI-Induced Cardiac Dysfunction and Remodeling

**Figure 2:**
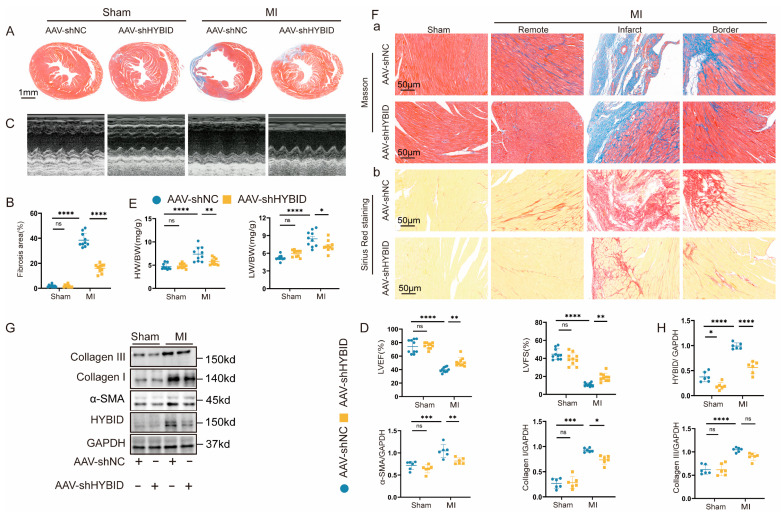
HYBID knockdown in cardiac tissue mitigates the fibrotic response following MI. The mice were randomly assigned to either the MI group or the sham operation group following injection with adeno-associated virus serotype 9 (AAV9) encoding HYBID (AAV-shHYBID) or a negative control (AAV-shNC). (**A**,**B**) Representative images of Masson staining and quantification of the hearts from the four groups of mice (n = 10). (**C**,**D**) Representative M-mode echocardiography images and analysis in the four groups of mice (n = 10). (**E**) Heart weight (HW)/body weight (BW) and lung weight (LW)/BW ratios of each group (n = 10). (**F**) Representative Masson staining (Panel a) and Sirius red staining (Panel b) (n = 10). (**G**,**H**) Representative Western blot images and quantification of HYBID, collagen I, collagen III, and α-SMA protein levels in the four groups (n = 6). * *p* < 0.05, ** *p* < 0.01, *** *p* < 0.001, and **** *p* < 0.0001. (**B**,**D**,**E**,**H**) by two-way ANOVA followed by Tukey’s multiple comparisons test. LVEF: left ventricular ejection fraction; and FS: fraction shortening.

We developed a fibroblast-targeted AAV9 system featuring FSP1 promoter-driven expression of HYBID-targeting shRNA to specifically examine HYBID’s role in myocardial fibrosis and ventricular remodeling [28,29]. This construct was administered via tail vein injection to specifically silence HYBID expression in cardiac fibroblasts of mice. The efficacy of HYBID knockdown was confirmed at both the mRNA and protein levels using qPCR and Western blot analyses, respectively (Figure S2B and Figure 2G,H). AAV-treated mice were subjected to MI/sham surgery at two weeks post-injection. Echocardiography revealed progressive ventricular dilation in MI groups by 14 weeks old (Figure S2A), with tissue analysis confirming fibrotic remodeling.

Following MI, a pronounced accumulation of collagen and extensive myocardial fibrosis were observed in both the infarct and border zones of the heart (Figure 2A,B,F). Histological analysis revealed that the normal myocardium in the left ventricular wall was replaced by thin fibrotic scar tissue, indicative of severe cardiac remodeling. Echocardiographic measurements further supported these findings, showing a marked reduction in LVEF and LVFS (Figure 2C,D). The HW/BW and LW/BW ratios were significantly elevated in MI mice compared to sham controls (Figure 2E), demonstrating marked cardiac hypertrophy and pulmonary congestion secondary to ischemic injury. These results suggest that HYBID plays a critical role in promoting myocardial fibrosis and adverse cardiac remodeling post-MI. While no significant differences were observed at baseline, the AAV-shHYBID-MI group demonstrated significantly improved cardiac function compared to the AAV-shNC-MI group, as evidenced by enhanced LVEF and LVFS (Figure 2C–E). Histological analysis using Masson’s trichrome and Sirius red staining revealed a substantial reduction in collagen deposition in the AAV-shHYBID-MI group, particularly in the non-infarct area, compared to the control group (Figure 2F). These findings collectively suggest that HYBID knockdown attenuates pulmonary edema, cardiac remodeling, and the progression toward heart failure post-MI.

Further mechanistic investigation revealed that HYBID downregulation significantly reduced the expression of key fibrotic markers, including collagen I, collagen III, and α-smooth muscle actin (α-SMA), in the AAV-shHYBID-MI group compared to the AAV-shNC-MI group (Figure 2G,H). These results indicate that HYBID silencing suppresses fibroblast activation and ECM deposition, thereby mitigating myocardial fibrosis and adverse cardiac remodeling. These findings suggest that targeting HYBID could offer a promising strategy to improve outcomes in patients with MI and subsequent heart failure. By attenuating fibrosis and preserving cardiac function, HYBID inhibition may help delay or prevent the transition from compensatory remodeling to overt heart failure.

### 3.3. Overexpression of HYBID Exacerbates MI-Induced Cardiac Dysfunction and Remodeling

**Figure 3:**
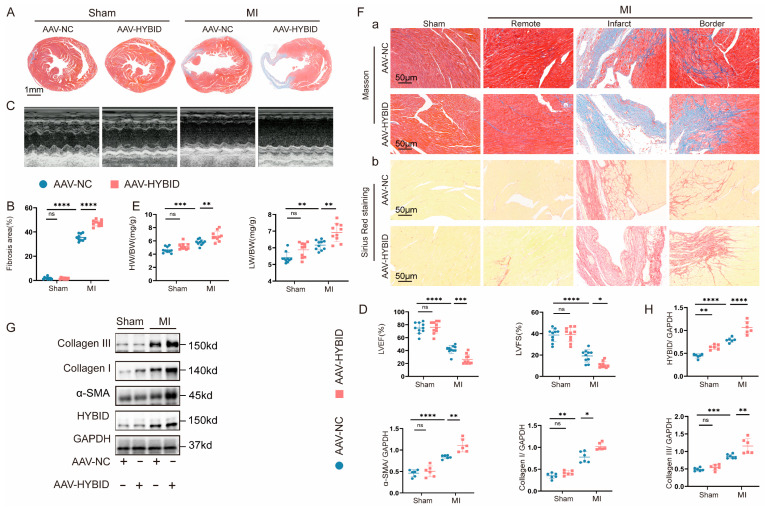
HYBID overexpression in cardiac tissue results in an exacerbated fibrotic response subsequent to MI. The mice were randomly assigned to either the MI group or the sham operation group following injection with AAV-HYBID or AAV-NC. (**A**,**B**) Representative images of Masson staining and quantification of the hearts from the four groups of mice (n = 10). (**C**,**D**) Representative M-mode echocardiography images and analysis from each group (n = 10). (**E**) HW/BW and LW/BW ratios of each group (n = 10). (**F**) Representative Masson staining (Panel a) and Sirius red staining (Panel b) (n = 10). (**G**,**H**) Representative Western blot images and quantitation of HYBID, collagen I, collagen III, and α-SMA protein levels in the four groups of mice (n = 6). * *p* < 0.05, ** *p* < 0.01, *** *p* < 0.001, and **** *p* < 0.0001. (**B**,**D**,**E**,**H**) by two-way ANOVA followed by Tukey’s multiple comparisons test.

To further elucidate the role of HYBID in myocardial fibrosis and cardiac remodeling, we specifically overexpressed HYBID in cardiac fibroblasts using AAV9 under the FSP1 promoter. The efficiency of HYBID overexpression was confirmed at both the mRNA and protein levels by qPCR (Figure S2C) and Western blot analysis (Figure 3G,H). Macroscopic examination of the ventricles revealed that HYBID overexpression significantly exacerbated cardiac remodeling and fibrosis following MI (Figure 3A,B). Mice in the AAV-HYBID-MI group exhibited higher HW/BW and LW/BW ratios compared to the AAV-NC-MI group, indicating aggravated cardiac hypertrophy and pulmonary congestion (Figure 3E). Echocardiographic analysis further demonstrated that HYBID overexpression markedly impaired cardiac function, as evidenced by reduced LVEF and LVFS (Figure 3C,D). Histological assessment using Masson’s trichrome and Sirius red staining revealed that HYBID overexpression intensified myocardial fibrosis and collagen deposition, particularly in the border and remote zones of the infarcted hearts, compared to the control group (AAV-NC-MI) (Figure 3F). Consistent with these findings, Western blot analysis showed that HYBID overexpression significantly upregulated the expression of key fibrotic markers, including collagen I, collagen III, and α-SMA (Figure 3G,H). Collectively, these results demonstrate that HYBID overexpression exacerbates cardiac fibrosis, ventricular remodeling, and functional deterioration following MI.

### 3.4. HYBID Modulates Cardiac Fibroblast Activation In Vitro

**Figure 4:**
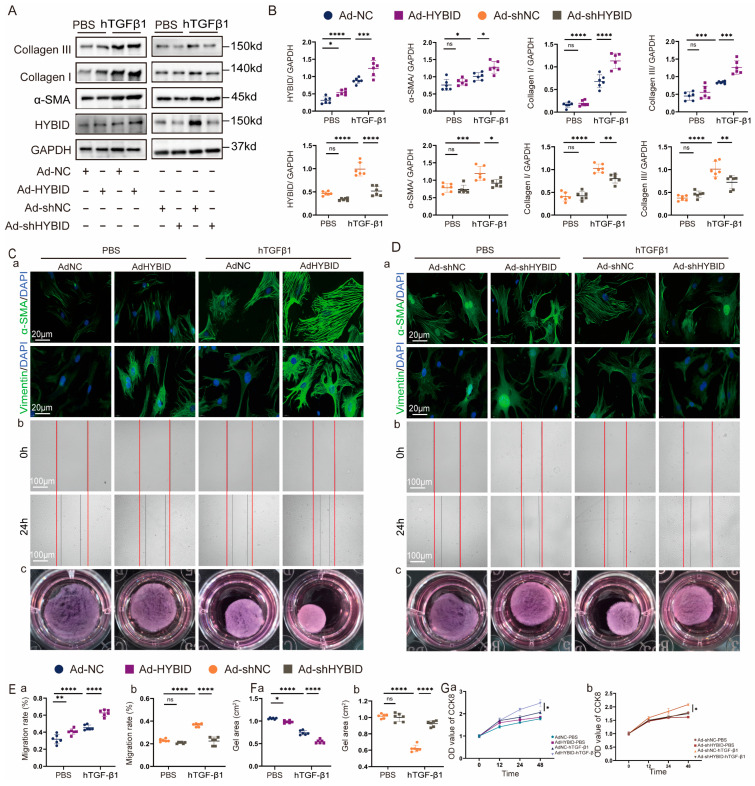
HYBID modulates hTGF-β1-induced cardiac fibroblast activation in vitro. Following transfection with adenoviruses specifically designed for the overexpression and silencing of HYBID, the impact of HYBID on fibroblast activation induced by hTGF-β1 was evaluated. (**A**,**B**) Western blot and quantification of HYBID, collagen I, collagen III, and α-SMA protein levels in CFs transfected with Ad-NC or Ad-HYBID and then treated with PBS or hTGF-β1 for 24 h (n = 6) or infected with indicated adenovirus (n = 6). (**C**,**D**) Representative images of immunofluorescence staining (a), scratch the wounds (b), and collagen gel contraction (c) in CFs infected with indicated adenovirus and then treated with PBS or hTGF-β1 for 24 h (n = 6). The red lines mark the scratch edges. (**E**) Quantitative analysis of scratch migration assays in CFs after HYBID overexpression (a) and knockdown (b) (n = 6). (**F**) Quantitative analysis of gel contraction assays in CFs after HYBID overexpression (a) and knockdown (b) (n = 6). (**G**) Quantitative analysis of cell proliferation assays (CCK-8) in CFs after HYBID overexpression (a) and knockdown (b) (n = 6). * *p* < 0.05, ** *p* < 0.01, *** *p* < 0.001, and **** *p* < 0.0001. (**B**,**E**–**G)** by two-way ANOVA followed by Tukey’s multiple comparisons test.

To investigate the role of HYBID in CF activation, CFs were isolated from neonatal mouse hearts and stimulated with hTGF-β1, a well-established profibrotic cytokine, to simulate the pathological processes underlying fibrotic diseases in vitro [30]. Following hTGF-β1 stimulation, CFs transfected with control adenovirus (Ad-NC or Ad-shNC) exhibited significantly elevated expression levels of HYBID, myofibroblast markers (α-SMA and vimentin), and fibrotic markers (collagen I and III) (Figure 4A–D). These findings suggest that hTGF-β1 induces a profibrotic phenotype in CFs, characterized by increased HYBID expression and enhanced fibroblast activation.

To further characterize the functional consequences of hTGF-β1 stimulation, we performed a series of in vitro assays. Scratch migration assays revealed enhanced migratory capacity of CFs, while CCK8 assays demonstrated increased proliferative activity following hTGF-β1 treatment (Figure 4C,D, plan b and Figure 4E,G). Additionally, collagen gel contraction assays and immunofluorescence staining of α-SMA and vimentin confirmed that hTGF-β1 promotes CF transdifferentiation into myofibroblasts, a key step in fibrotic remodeling (Figure 4D, plan c and Figure 4F). These results collectively indicate that hTGF-β1 drives CF activation, migration, proliferation, and myofibroblast differentiation, all of which are critical processes in the development of myocardial fibrosis.

To elucidate the specific role of HYBID in these processes, CFs were transfected with either HYBID-silencing adenovirus (Ad-shHYBID) or HYBID-overexpressing adenovirus (Ad-HYBID). Overexpression of HYBID significantly amplified the effects of hTGF-β1, further elevating the expression of collagen I, collagen III, and α-SMA (Figure 4A,B). Functionally, HYBID overexpression enhanced hTGF-β1-induced collagen gel contraction, CF migration, proliferation, and myofibroblast transdifferentiation (Figure 4C–F). Conversely, HYBID silencing effectively attenuated these profibrotic responses, reducing collagen production, inhibiting CF migration and proliferation, and suppressing myofibroblast differentiation (Figure 4A–G). These findings demonstrate that HYBID is a critical regulator of hTGF-β1-induced CF activation and collagen synthesis.

### 3.5. RNA-Seq Reveals MMP13 as a Key Downstream Target in HYBID-Mediated Ventricular Remodeling

**Figure 5:**
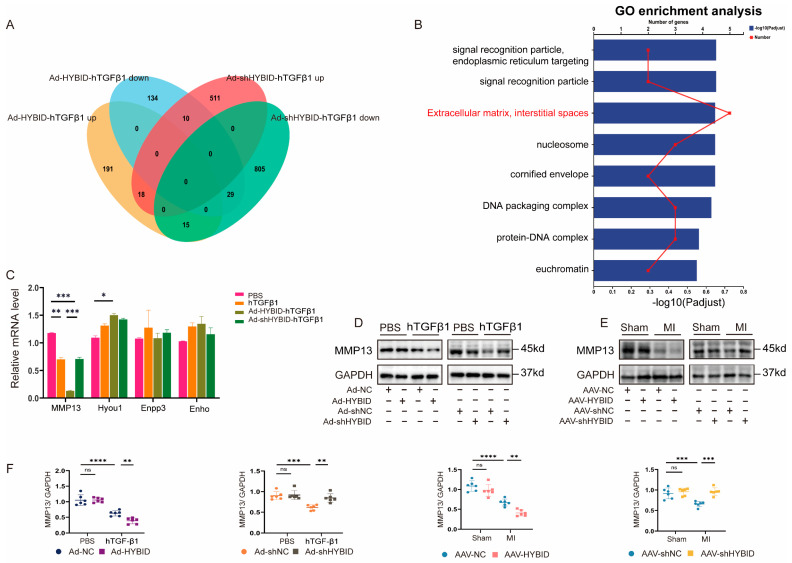
RNA-seq indicates that MMP13 is involved in the regulation of ventricular remodeling by HYBID. (**A**) Venn diagram of RNA-seq analysis performed in CFs with adenovirus to silence or overexpress HYBID (n = 3) (log2(Fold-change) > 2, adjusted *p*-value < 0.05). (**B**) GO term enrichment analysis of 25 genes upregulated in four groups (log2(Fold-change) > 2, adjusted *p*-value < 0.05). (**C**) Analysis of Enpp3, Mmp13, Hyou1, and Enho mRNAs in different groups (n = 6). (**D**) Representative Western blot images of MMP13 protein expression in HYBID-silenced and HYBID-overexpressed CFs after treatment with hTGF-β1 or PBS (n = 6). (**E**) Representative Western blot images of MMP13 protein expression in HYBID-silenced and HYBID-overexpressed after MI (n = 6). (**F**) The quantification of MMP13 protein expression after in vitro and in vivo regulation of HYBID. * *p* < 0.05, ** *p* < 0.01, *** *p* < 0.001, and **** *p* < 0.0001. (**C**–**F**) by two-way ANOVA followed by Tukey’s multiple comparisons test. (**B**) Adjusted *p*-values (Benjamini–Hochberg corrected; FDR < 0.05).

RNA-seq profiling of CFs under HYBID perturbation (knockdown vs. overexpression) with hTGF-β1 co-stimulation revealed key pathways in fibrosis regulation. Comparative analysis of the RNA-seq datasets revealed significant transcriptional changes associated with HYBID modulation. Specifically, in CFs overexpressing HYBID (Ad-HYBID), 224 genes were significantly upregulated, while 173 genes were downregulated compared to the control group (Ad-NC). Conversely, in CFs with HYBID knockdown (Ad-shHYBID), 539 genes were upregulated, and 849 genes were downregulated relative to the control group (Ad-shNC) (Figure S3A–C). Further analysis identified 10 genes that were upregulated in the Ad-shHYBID group but downregulated in the Ad-HYBID group as well as 15 genes that exhibited the opposite pattern (Figure 5A). GO analysis revealed that these differentially expressed genes were significantly enriched in pathways related to ECM organization and extracellular structural organization, both of which are critically involved in cardiac remodeling (Figure 5B). Among the ECM-related genes, we focused on Enpp3, Enho, Hyou1, and MMP13 due to their potential roles in fibrosis and remodeling. Notably, MMP13 emerged as a key downstream target of interest (Figure 5C). MMP13, a matrix metalloproteinase, is known to play a pivotal role in ECM remodeling and collagen regulation within atherosclerotic plaques, making it a compelling candidate for further investigation [20].

To validate our RNA-seq results, we examined MMP13 protein expression in CFs following HYBID knockdown or overexpression. Consistent with the sequencing data, HYBID overexpression significantly reduced MMP13 expression, whereas HYBID knockdown led to its upregulation (Figure 5D). This trend was further corroborated in vivo, where MMP13 expression levels in MI mice mirrored the changes observed in vitro following HYBID modulation (Figure 5E). These results suggest that MMP13 suppression may contribute to the exacerbation of myocardial fibrosis, highlighting its potential role as a mediator of HYBID-dependent fibrotic responses.

### 3.6. HYBID Regulates the Activation of Fibroblasts Through MMP13

**Figure 6:**
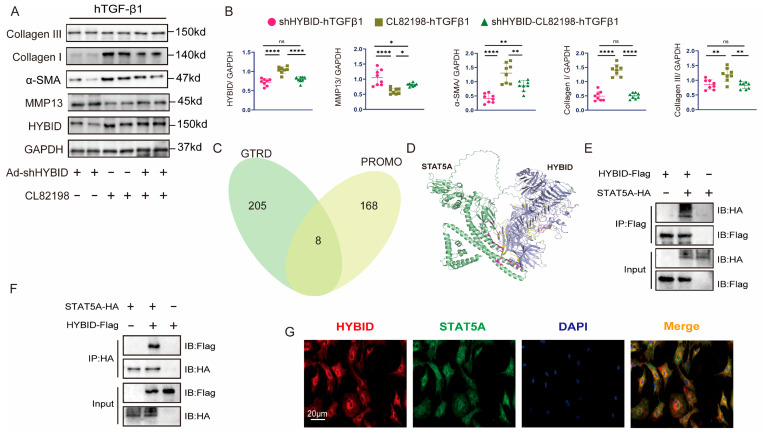
HYBID regulates fibroblast activation and pathological cardiac fibrosis through MMP13. (**A**,**B**) Western blot and quantification of collagen I and III, α-SMA, HYBID, and MMP13 protein levels in CFs transfected with Ad-shHYBID or an MMP13 inhibitor (CL82198) and then treated with hTGF-β1 (n = 8). (**C**) Venn diagram of the MMP13 transcription factors predicted using the PROMO and CTRD databases. (**D**) Structure-based interaction surface of HYBID and the STAT5A model by AlphaFold3. (**E**) IP with Flag-HYBID and IB with HA-STAT5A in 293T cells. (**F**) IP with HA-STAT5A and IB with Flag-HYBID in 293T cells. (**G**) Immunofluorescence staining for co-localization of HYBID and STAT5A in CFs (n = 3). Scale bar: 20 μm. * *p* < 0.05, ** *p* < 0.01, and **** *p* < 0.0001. (**B**,**E**–**G**) by two-way ANOVA followed by Tukey’s multiple comparisons test.

CFs were assigned to three groups: (i) Ad-shHYBID (MOI = 50), (ii) CL82198 (10 μM) [31], and (iii) Ad-shHYBID+CL82198. After 24 h pretreatment, all groups received hTGF-β1 (10 ng/mL, 24 h) to induce fibrosis. As anticipated, the group treated with the MMP13 inhibitor exhibited elevated levels of fibrosis compared to the Ad-shHYBID group (Figure 6A,B). Functional assays, including collagen gel contraction, cell proliferation, migration, and transdifferentiation into myofibroblasts, further supported these findings (Figure S4A–D). Notably, the concurrent administration of the MMP13 inhibitor with Ad-shHYBID transfection significantly attenuated the anti-fibrotic effects of HYBID silencing, without altering HYBID expression levels (Figure 6A–E and Figure S4A–D). These results suggest that MMP13 acts as a critical downstream mediator of HYBID in regulating fibroblast activation and myocardial fibrosis.

### 3.7. HYBID Inhibits STAT5A-Mediated MMP13 Transcription in Cardiac Fibroblasts

To delineate the transcriptional regulation of HYBID-dependent myocardial remodeling post-MI, we performed computational prediction of MMP13-regulating transcription factors. Using the PROMO algorithm (v3.0.2) and Genomic Transcription Factors Database (GTRD), we identified eight candidate transcription factors with high-probability binding sites in the MMP13 promoter region (Figure 6C). These factors were prioritized based on binding site conservation across species, motif score (>0.95), and established roles in cardiac fibrosis pathways. Sequence alignment analysis via the JASPAR database further refined these predictions, revealing that signal transducer and activator of transcription 5A (STAT5A) exhibited the highest binding affinity among the candidates (Supplementary Table S3). STAT5A has been previously implicated in ischemic heart disease, making it a compelling candidate for further investigation [32]. Subsequent mRNA validation in CFs confirmed that STAT5A expression was significantly downregulated following hTGF-β1 stimulation (Figure S5A), suggesting its potential role in post-MI remodeling. In vitro experiments demonstrated that HYBID overexpression significantly downregulated STAT5A expression, whereas HYBID knockdown led to its upregulation (Figure S5B). This pattern was consistent with observations in MI mice, where STAT5A expression levels mirrored changes in HYBID modulation (Figure S5C).

Protein–protein interactions were predicted using AlphaFold3 (v3.0) with default parameters (Figure 6D). The top-ranked HYBID–STAT5A complex (pLDDT > 85) exhibited favorable binding energy (−17.0 kcal/mol), with interacting residues validated by COIP. COIP experiments in HEK293-T cells confirmed the physical interaction between exogenous STAT5A and HYBID, demonstrating reciprocal binding (Figure 6E,F). Furthermore, confocal microscopy revealed co-localization of HYBID and STAT5A in the cytoplasm of CFs, supporting their functional interaction (Figure 6G). The co-localization of these proteins in the cytoplasm further suggests that HYBID may sequester STAT5A, preventing its nuclear translocation and subsequent activation of MMP13 transcription. These findings suggest that HYBID may modulate MMP13 transcription by competitively binding to STAT5A, thereby inhibiting its transcriptional activity.

**Figure 7:**
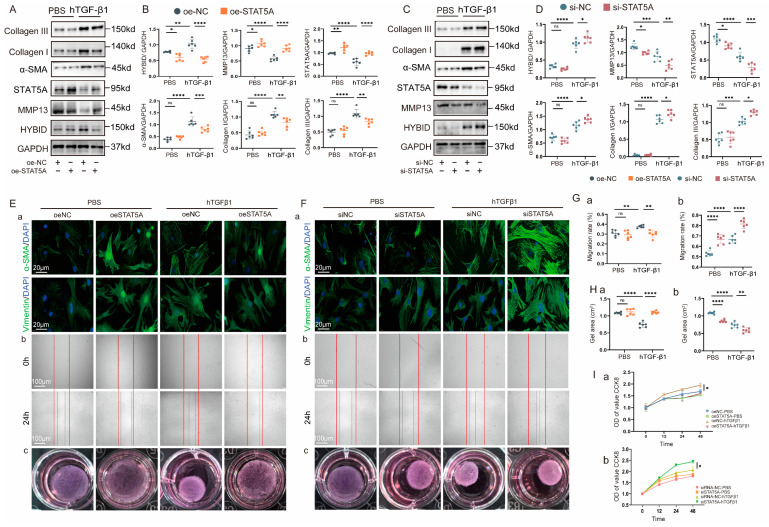
HYBID inhibits STAT5A-mediated MMP13 transcription in cardiac fibroblasts. Effects of STAT5A overexpression and silencing on hTGF-β1-induced fibroblast activation in vitro. (**A**,**B**) Western blot and quantification of HYBID, MMP13, STAT5A, collagen I and III, and α-SMA protein levels in CFs transfected with STAT5A overexpression plasmids and then treated with hTGF-β1 (n = 6). (**C**,**D**) Western blot and quantification of HYBID, MMP13, STAT5A, collagen I and III, and α-SMA protein levels in CFs transfected with STAT5A siRNA and then treated with hTGF-β1 (n = 6). (**E**,**F**) Representative images of immunofluorescence staining (a), scratch migration assay (b), and collagen gel contraction assay (c) in CFs transfected with plasmid or siRNA, followed by treatment with PBS or hTGF-β1 for 24 h (n = 6). Red lines indicate scratch wound margins. (**G**) Quantitative analysis of scratch migration assays in each group after STAT5A overexpression (a) and silencing (b) (n = 6). (**H**) Quantitative analysis of gel contraction assays in fibroblasts after STAT5A overexpression (a) and silencing (b) (n = 6). (**I**) Quantitative analysis of CCK8 in fibroblasts after STAT5A overexpression (a) and silencing (b) (n = 6). (**B**,**E**–**G**) ns *p* >0.05, * *p* < 0.05, ** *p* < 0.01, *** *p* < 0.001, and **** *p* < 0.0001. (**B**) by two-way ANOVA followed by Tukey’s multiple comparisons test.

To further elucidate the regulatory role of STAT5A in HYBID-mediated CF differentiation, we transfected CFs with STAT5A plasmids or siRNA (Figure S6A). Overexpression of STAT5A resulted in a significant upregulation of MMP13 and STAT5A, alongside a downregulation of HYBID expression (Figure 7A,B). Functionally, STAT5A overexpression attenuated collagen synthesis, gel contraction, myofibroblast transformation, cell migration, and proliferation (Figure 7E–G, plan a). Conversely, STAT5A knockdown exacerbated these fibrotic responses (Figure 7C–G, plan b). These results suggest that HYBID suppresses MMP13 expression by inhibiting STAT5A transcriptional activity, thereby promoting myocardial fibrosis.

### 3.8. STAT5A-Mediated HYBID-Regulated Pathological Cardiac Remodeling

**Figure 8:**
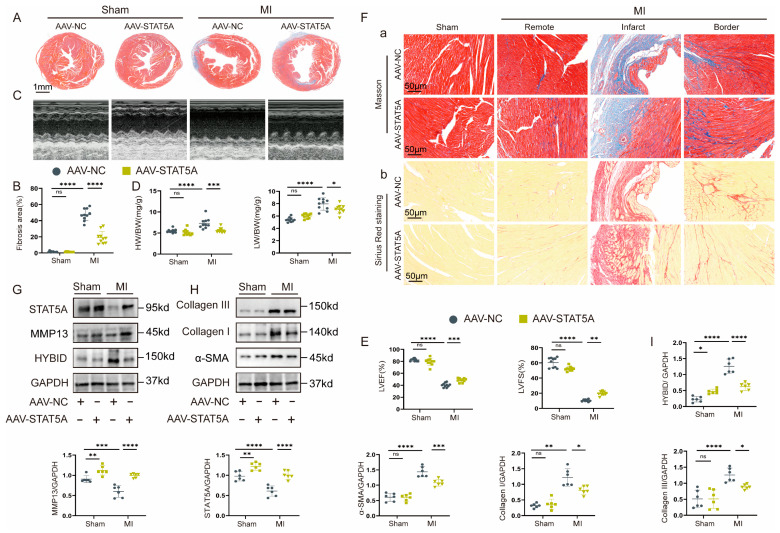
STAT5A overexpression in cardiac tissue mitigates the fibrotic response following MI. The mice were randomly assigned to either the MI group or the sham operation group following injection with AAV9 encoding STAT5A (AAV-STAT5A) or a negative control (AAV-NC). (**A**,**B**) Representative images of Masson staining and quantification of the hearts from the four groups of mice (n = 10). (**C**,**D**) Representative M-mode echocardiography image and analysis of the four groups of mice (n = 10). (**E**) HW/BW and LW/BW ratios of each group (n = 10). (**F**) Representative Masson staining (Panel a) and Sirius red staining (Panel b) (n = 10). (**G**–**I**) Representative Western blot images and quantification of HYBID, MMP13, STAT5A, collagen I, collagen III, and α-SMA protein levels in the four groups of mice (n = 6). * *p* < 0.05, ** *p* < 0.01, *** *p* < 0.001, and **** *p* < 0.0001. **B**,**D**,**E**,**I** by two-way ANOVA followed by Tukey’s multiple comparisons test.

The AAV9-FSP1-STAT5A construct was generated by cloning full-length mouse STAT5A cDNA downstream of the 1.2 kb FSP1 promoter. Viruses were packaged and purified with titers determined by qPCR. Male C57BL/6 mice received intravenous injections 3 days pre-MI, with controls receiving AAV9-FSP1-GFP. The efficiency of STAT5A overexpression was confirmed at both the mRNA and protein levels (Figure S6B and Figure 8G–I). Consistent with our expectations, the AAV-STAT5A-MI group exhibited significantly lower HW/BW and LW/BW ratios compared to the control group (AAV-NC-MI), indicating reduced cardiac hypertrophy and pulmonary congestion (Figure 8D). Echocardiographic analysis further revealed improved cardiac function in the AAV-STAT5A-MI group, as evidenced by enhanced LVEF and LVFS (Figure 8C,E). Histological assessment demonstrated that STAT5A overexpression attenuated myocardial fibrosis and collagen deposition in both the border and remote zones of the infarcted heart (Figure 8A,B,F). These findings were corroborated by Western blot analysis, which showed reduced expression of fibrotic markers in the AAV-STAT5A-MI group (Figure 8G–I).

**Figure 9:**
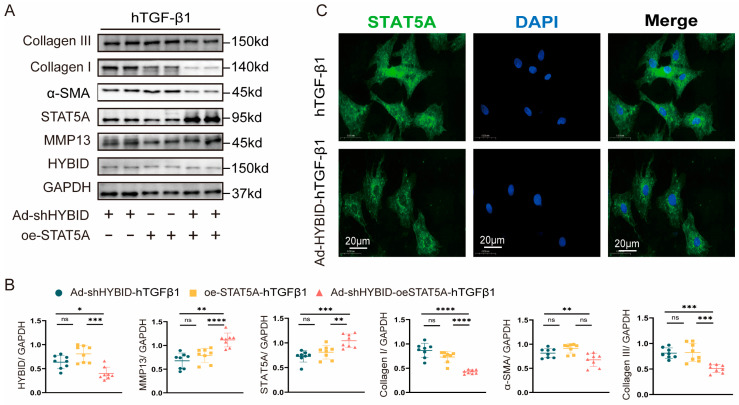
STAT5A interacts with HYBID to regulate pathological cardiac remodeling after MI. (**A**,**B**) Western blot and quantification of collagen I and III, α-SMA, STAT5A, HYBID, and MMP13 protein levels in CFs transfected with Ad-shHYBID or STAT5A overexpression plasmids and then treated with hTGF-β1 (n = 6). (**C**) STAT5A nuclear accumulation was determined using immunofluorescence staining in CFs transfected with Ad-HYBID (n = 3). The green area represents STAT5A, and the nuclei are blue. The error bars are the SD. ns *p* >0.05, * *p* < 0.05, ** *p* < 0.01, *** *p* < 0.001, and **** *p* < 0.0001. (**B**) by two-way ANOVA followed by Tukey’s multiple comparisons test.

To further investigate the synergistic effects of STAT5A overexpression and HYBID knockdown, we co-transfected CFs with Ad-shHYBID and STAT5A plasmids. Under hTGF-β1 stimulation, this combined approach resulted in a significant additive inhibition of fibrotic protein expression and cellular functions, including collagen synthesis, gel contraction, myofibroblast transformation, migration, and proliferation (Figure 9A,B and Figure S7). These results suggest that STAT5A overexpression and HYBID silencing act synergistically to suppress excessive fibroblast activation and fibrotic remodeling.

Finally, we explored the mechanism by which HYBID regulates STAT5A activity. Immunofluorescence staining revealed that Ad-HYBID treatment significantly reduced the nuclear import of STAT5A (Figure 9C). This finding suggests that HYBID binds to STAT5A in the cytoplasm, preventing its translocation to the nucleus and thereby inhibiting MMP13 transcription. This mechanism likely contributes to the facilitation of fibroblast activation and pathological cardiac remodeling.

## 4. Discussion

In this study, we identified HYBID as a novel regulator of pathological cardiac remodeling, a critical process underlying heart failure progression. Our findings revealed that HYBID expression was significantly upregulated in MI-induced mice, particularly in CFs but not in CMs. This cell-type-specific upregulation suggests a unique role of HYBID in fibroblast activation and myocardial fibrosis, which are central to adverse cardiac remodeling. By employing AAV-shHYBID and AAV-HYBID viral constructs, we demonstrated that HYBID knockdown ameliorated MI-induced cardiac dysfunction and remodeling, whereas HYBID overexpression exacerbated these pathological changes. These results collectively highlight HYBID as a key mediator of fibroblast-to-myofibroblast transformation and its pivotal role in post-MI cardiac remodeling. Mechanistically, we uncovered that HYBID interacts with STAT5A to suppress the transcriptional activity of the *MMP13* promoter, thereby promoting the transition of fibroblasts into myofibroblasts. This finding provides a previously unrecognized molecular pathway linking HYBID to ECM remodeling and fibrosis. The downregulation of MMP13, a matrix metalloproteinase involved in ECM degradation, likely contributes to the accumulation of fibrotic tissue and the stiffening of the ventricular wall, both hallmarks of heart failure. Our study thus adds a new layer of understanding to the complex regulatory network governing fibroblast activation and myocardial fibrosis.

Fibrotic remodeling is a hallmark of adverse cardiac outcomes, with its extent strongly correlating with the severity of functional deficits in both clinical patients and experimental animal models [5]. The restoration of infarcted myocardium and preservation of ventricular structural integrity are critically dependent on fibroblast activation. Central to this process is the transdifferentiation of CFs into myofibroblasts, a pivotal event in modulating fibrotic responses. These activated myofibroblasts serve as the primary source of ECM proteins, driving the pathological remodeling observed in heart failure [2,3,4]. Understanding the mechanisms underlying myofibroblast activation is therefore essential for developing targeted therapeutic strategies to mitigate cardiac remodeling and its associated functional impairments.

RNA-seq analysis of mice two weeks post-MI revealed a significant upregulation of HYBID expression. Although HYBID has been implicated in the progression of myocardial fibrosis [15], the precise mechanisms remain poorly understood. In this study, we demonstrated that HYBID knockdown alleviated MI-induced cardiac dysfunction and fibrosis, thereby improving pathological remodeling, while HYBID overexpression exacerbated these effects. These findings position HYBID as a critical regulator of fibrotic processes post-MI. Notably, therapeutic strategies targeting cardiac fibrosis have the potential to reduce acute-phase mortality by preventing myocardial rupture and attenuating maladaptive remodeling of the distal myocardium [33]. Interestingly, while HYBID silencing significantly improved myocardial fibrosis, it had minimal impact on post-MI mortality in mice, suggesting that HYBID knockdown specifically targets fibrosis without compromising infarct healing. Furthermore, we observed that HYBID promotes diffuse fibrosis in the distal myocardium, a phenomenon commonly associated with chronic heart diseases such as aortic stenosis [34], myocardial hypertrophy [35], hypertensive heart disease, and systemic conditions linked to heart failure [36,37]. HYBID, located on human chromosome 15q25.1, encodes a signal peptide of 30 amino acids and features structural domains including one G8 domain, two GG domains, four PbH1 repeat sequences, and seven glycosylation sites [38,39]. Despite its significant impact on myocardial remodeling, the expression patterns of HYBID across different cardiac cell populations and remodeling contexts remain incompletely characterized. Our in vitro experiments revealed that HYBID is highly expressed in CFs, highlighting the need for systematic investigation into its role and mechanisms within these cells. These findings underscore the potential relevance of HYBID in a broad spectrum of fibrotic cardiac conditions, although further research is needed to validate these hypotheses.

TGF-β is a well-established profibrotic factor that stimulates collagen production and fibroblast-to-myofibroblast transition [40]. Consistent with previous studies, we found that HYBID expression is upregulated by TGF-β signaling [41]. CFs overexpressing HYBID exhibited heightened sensitivity to the profibrotic effects of hTGF-β1, whereas HYBID knockdown attenuated collagen release and myofibroblast differentiation. Excessive fibroblast activation and ECM deposition are key drivers of heart failure progression post-MI [42]. Our study further revealed that HYBID co-localizes with vimentin in the cytoplasm of CFs following hTGF-β1 treatment, emphasizing its role in facilitating the transition of CFs into myofibroblasts. These findings suggest that HYBID enhances collagen synthesis, cellular contraction, and ECM accumulation, thereby promoting fibrotic remodeling.

RNA-seq analysis identified MMP13 as a downstream target of HYBID. MMP13 plays a protective role in ischemic heart failure by promoting angiogenesis and myocyte adhesion, thereby enhancing mesenchymal stromal cell function [43]. HYBID depletion suppresses MMP13 expression in an IL1β-dependent manner [21]. Our results corroborate these findings, showing reduced MMP13 levels with HYBID overexpression and increased levels with HYBID knockdown. MMP13 is critical for collagen degradation within atherosclerotic lesions, preventing aberrant ECM remodeling [20,44]. In this study, HYBID silencing downregulated collagen synthesis, fibroblast transformation, and cellular functions such as proliferation and migration, effects that were reversible upon MMP13 inhibition. These results provide novel insights into the role of MMP13 in HYBID-regulated fibroblast differentiation.

STAT5A is a key transcriptional cofactor implicated in fibrotic diseases [45]. Its activation suppresses MMP2 and MMP9, which are involved in ECM degradation and tumor cell invasion [46]. MMP13 further facilitates ECM degradation by activating MMP2 and MMP9 [47,48]. We observed that MMP13 expression in CFs is positively regulated by STAT5A, and for the first time, we demonstrated an interaction between HYBID and STAT5A using protein docking, co-localization, and COIP experiments. Notably, HYBID overexpression reduced the nuclear import of STAT5A, suggesting that HYBID exacerbates cardiac remodeling by downregulating STAT5A activity and modulating MMP13 transcription. These findings highlight the HYBID–STAT5A–MMP13 axis as a novel regulatory pathway in post-MI cardiac remodeling and identify potential therapeutic targets for cardiac fibrosis.

While our findings support the central role of HYBID in cardiac fibrosis, several limitations warrant consideration: (1) The role of HYBID in other cardiac cell types and systemic fibrosis post-MI remains unexplored; (2) validation in alternative models (e.g., pressure overload) and human samples is needed; and (3) only male mice and short-term (≤4 weeks) effects were examined. As a secreted protein, HYBID may contribute to systemic fibrosis post-MI, requiring further investigation. The relatively small sample size, although consistent with most preclinical research standards, may limit the statistical power to detect subtle phenotypic differences or sex-specific effects. Future studies should address these gaps through multi-model validation, sex-stratified analyses, and evaluation of HYBID-targeted therapies in humans.

## 5. Conclusions

In summary, our study identifies HYBID as a critical regulator of fibroblast-to-myofibroblast transition and pathological cardiac remodeling. By elucidating the HYBID–STAT5A–MMP13 axis, we provide a mechanistic framework for understanding how HYBID promotes fibrosis and adverse remodeling post-MI. These findings not only advance our understanding of the molecular mechanisms underlying heart failure but also highlight HYBID as a promising therapeutic target for preventing and treating cardiac fibrosis and remodeling. Future research should focus on translating these findings into clinical applications, offering new hope for patients with heart failure.

## Data Availability

The data that support the findings of this study are available in the Supplementary Materials of this article.

## Supplementary Materials

The following supporting information can be downloaded at https://www.mdpi.com/article/10.3390/biomedicines13061302/s1.
