# Supplementary material for: Hyaluronan-Binding Protein Promotes Fibroblast Transformation and Heart Failure by Modulating the STAT5A–MMP13 Pathway
Source: Biomedicines. 2025 May 26;13(6):1302. doi: 10.3390/biomedicines13061302 (PMC12189040; doi:10.3390/biomedicines13061302)
Supplement: Supplementary file 1 [file biomedicines-13-01302-s001.zip › biomedicines-3632897-supplementary.pdf]

HYBID Promotes Fibroblast Transformation and Heart Failure by Modulating the STAT5A-MMP13 Pathway

Hui Yan [1, 3,4, #], Bing Huang [2, 3,4, #], Bofang Zhang[2, 3,4], Yunyao Li[2, 3,4], Qiping zhou[1], Abudoureyimu Ayipali [1], Guiqiu Cao [1, *], Hong Jiang[1,2, 3,4, *]

1. Department of Cardiology, The Fifth Affiliated Hospital of Xinjiang Medical University, Urumqi, China.

2. Department of Cardiology, Renmin Hospital of Wuhan University, Wuhan, China.

3. Hubei Key Laboratory of Cardiology, Wuhan, China.

4. Cardiovascular Research Institute of Wuhan University, Wuhan, China.

[#] These authors contributed equally to this work.

Correspondence to: Hong Jiang, email: whuhong_jiang@163.com; Guiqiu Cao, email: caogq1215@163.com

This PDF file includes:

Materials and Methods

Figures S1 to S7

Tables S1 to S3

Figures S1 to S7

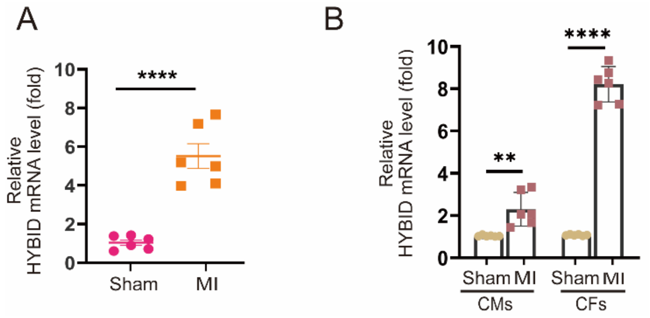

Figure S1 HYBID expression is increased in cardiac fibroblasts and mouse remodeling hearts. A The mRNA of HYBID levels in sham or MI group (n=6). B The mRNA of HYBID levels in isolated CMs and CFs at 4 weeks after MI or Sham (n=6). The Error bars are the SD. *P<0.05, ** P <0.01, *** P <0.001, and **** P <0.0001. **A, B** by two-tailed unpaired *Student's t*-test.

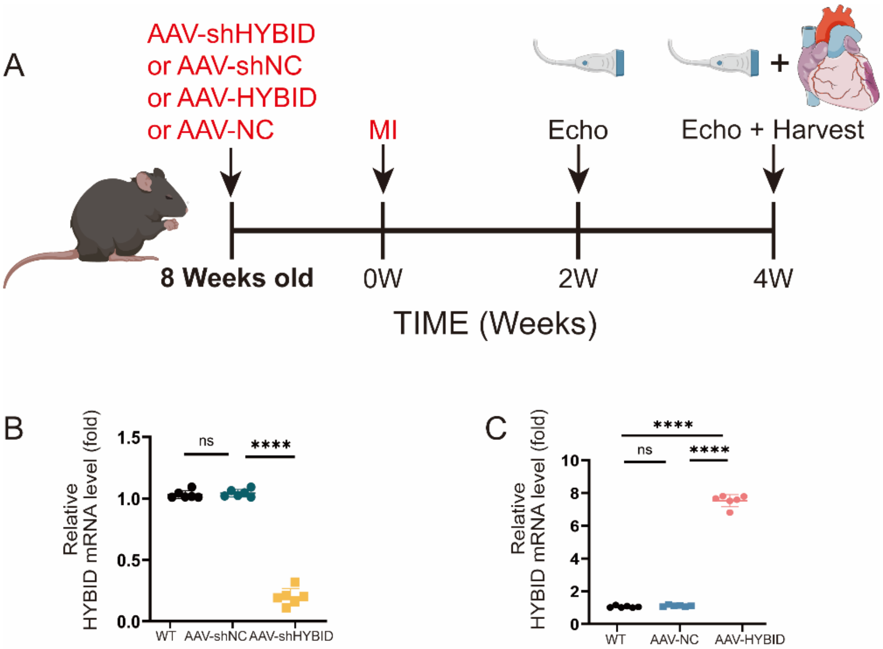

Figure S2 HYBID overexpression facilitates fibroblast activation and cardiac remodeling in mice with exacerbated apoptosis.

A Schematic for echocardiography and sample collection from 4 groups: Ad-shNC and Ad-shHYBID at 4 weeks after sham or MI. **B** Relative mRNA expression level of HYBID in the myocardium of mice injected with adeno-associated virus (AAV)-shHYBID or the negative control (NC), as detected by quantitative real-time PCR (n=6). **C** Relative mRNA expression level of HYBID in the myocardium of mice injected with AAV-HYBID or AAV-NC (n=6). The error bars are the SD. *P<0.05, **P<0.01, **P<0.001, and ****P<0.0001 **B-C** by two-way ANOVA followed by Tukey's multiple comparisons test.

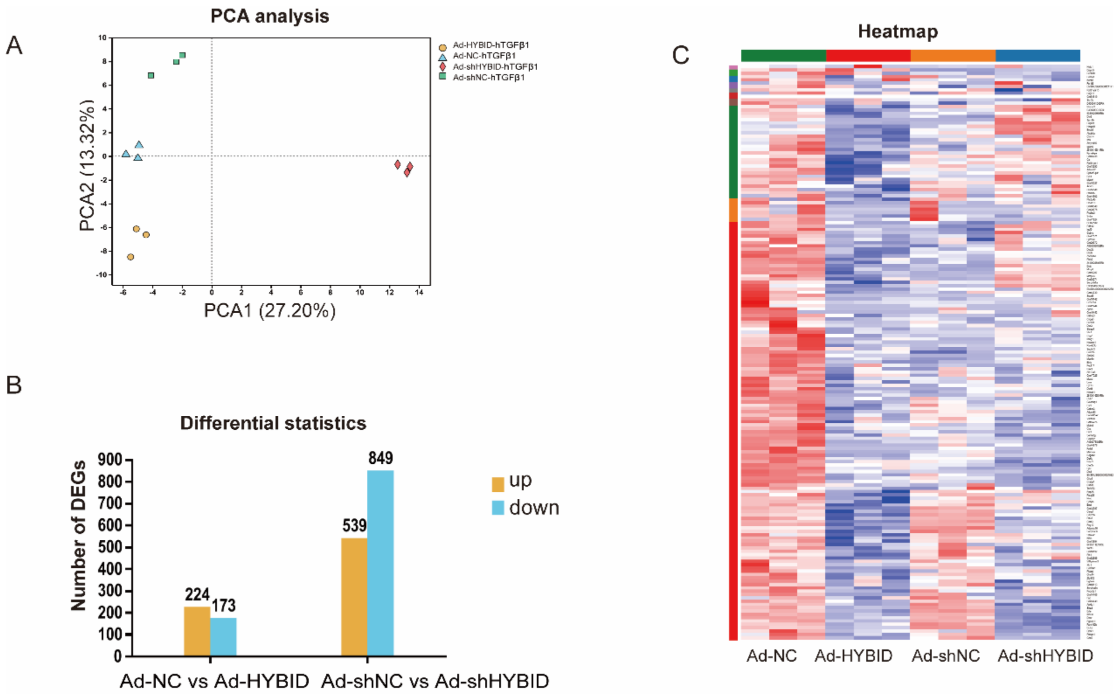

Figure S3 RNA-seq indicates that matrix metallopeptidase 13 (MMP13) is involved in the regulation of ventricular remodelling by HYBID. A PCA of each group of RNA-seq samples (n=3). **B** Differential gene analysis among the four groups. **C** Heatmap of differentially expressed genes in the four groups.

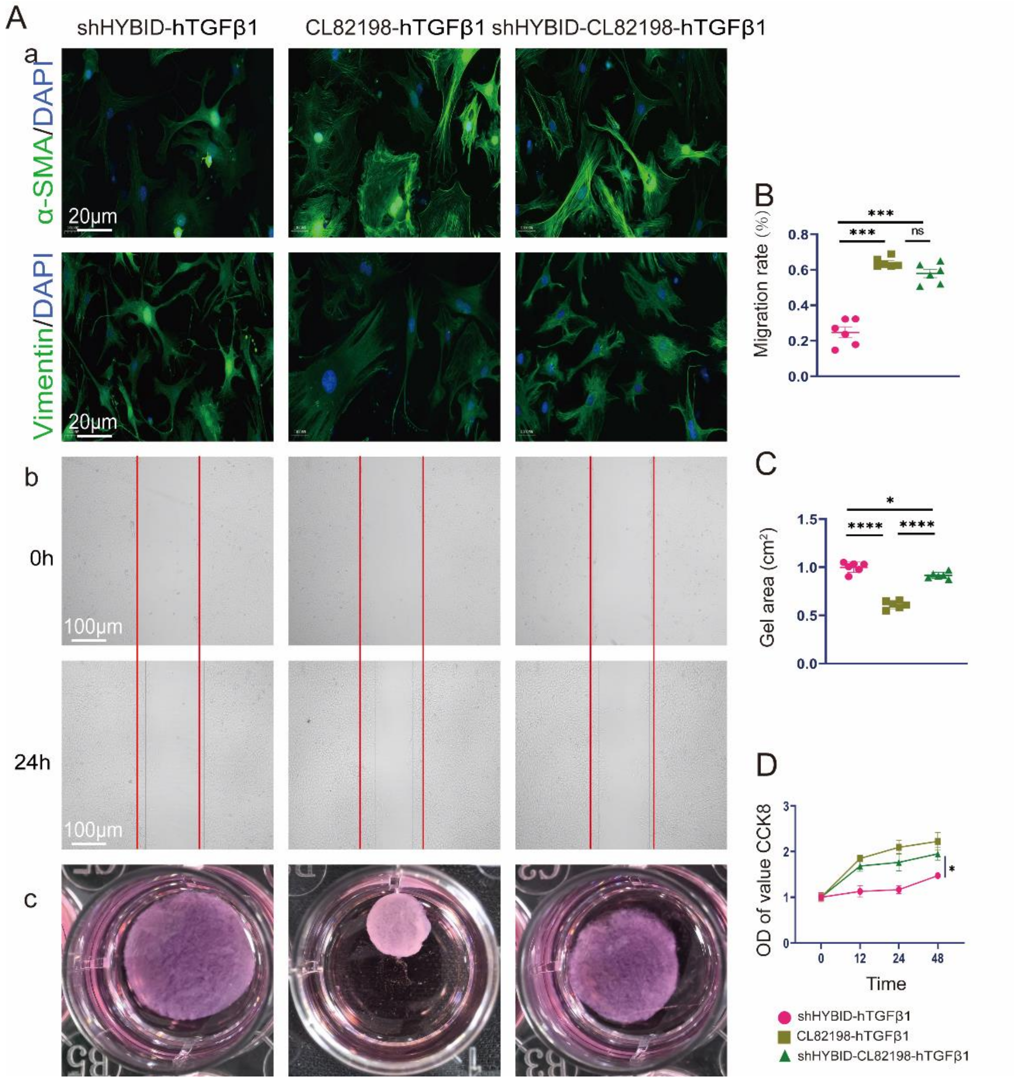

Figure S4 MMP13 inhibitors significantly reversed the beneficial effects of shHYBID on cellular functions. A Representative images of immunofluorescence staining (a), scratch the wounds (b) and collagen gel contraction (c) in CFs infected with indicated adenovirus and then treated with PBS or hTGF-β1 for 24 h (n=6). The red lines mark the scratch edges. **B-C** Quantification of migration distances and collagen gel contraction. **D** CCK8 assay to detect fibroblast proliferation. The error bars are the SD. *P<0.05, **P<0.01, **P<0.001, and ****P<0.0001 **B-D** by two-way ANOVA followed by Tukey’s multiple comparisons test.

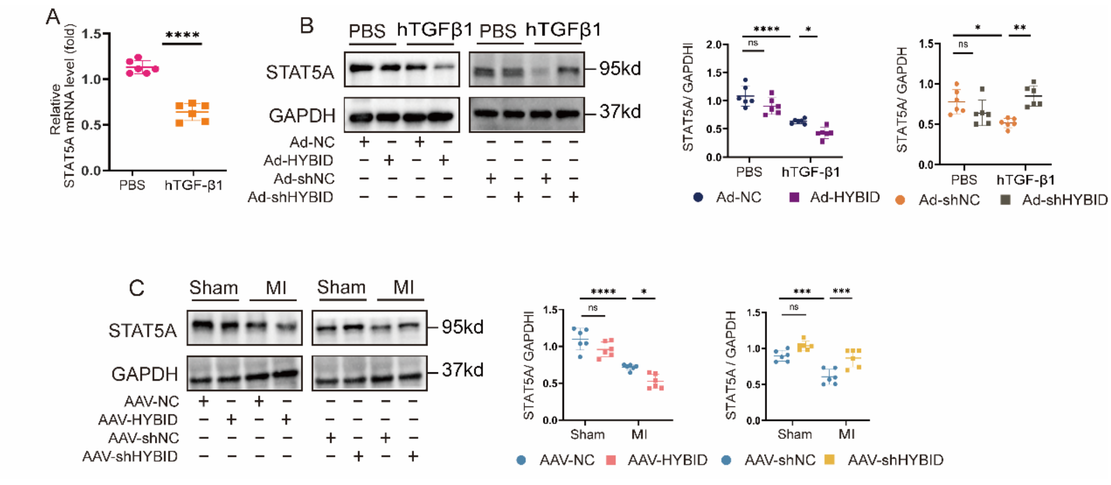

Figure S5 STAT5A is involved in HYBID-regulated pathological cardiac remodelling after MI. A Relative STAT5A mRNA expression levels in CFs. **B** Representative Western blot images and quantification of STAT5A proteins in HYBID-silenced and HYBID-overexpressing CFs after treatment with hTGF-β1 or PBS (n=6). **C** Representative Western blot images and quantification of the STAT5A protein in the myocardium of mice injected with AAV-HYBID or AAV-shHYBID (n=6). The error bars are the SD. *P<0.05, **P<0.01, **P<0.001, and ****P<0.0001 **A** by two-tailed unpaired Student's t-test. **B-C** by two-way ANOVA followed by Tukey's multiple comparisons test.

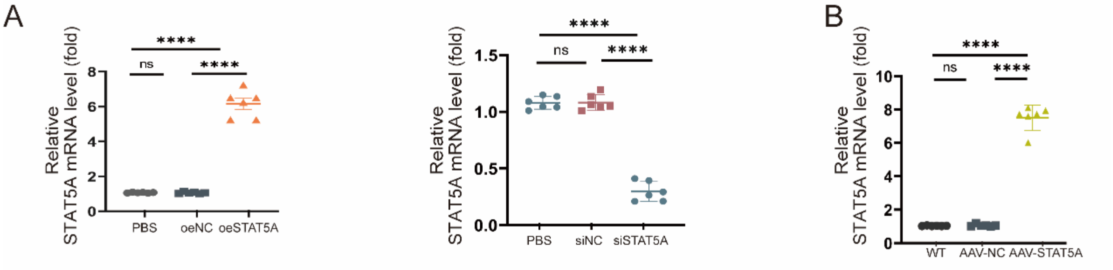

Figure S6 STAT5A overexpression in cardiac tissue mitigates the apoptosis response following MI.

A Relative STAT5A mRNA expression levels in CFs after transduction with STAT5A overexpression plasmids or STAT5A siRNA (n=6). **B** Relative STAT5A mRNA expression levels in the myocardium of mice injected with AAV-NC or AAV- STAT5A (n=6). The error bars are the SD. *P<0.05, **P<0.01, **P<0.001, and ****P<0.0001 **A-B** by two-way ANOVA followed by Tukey's multiple comparisons test.

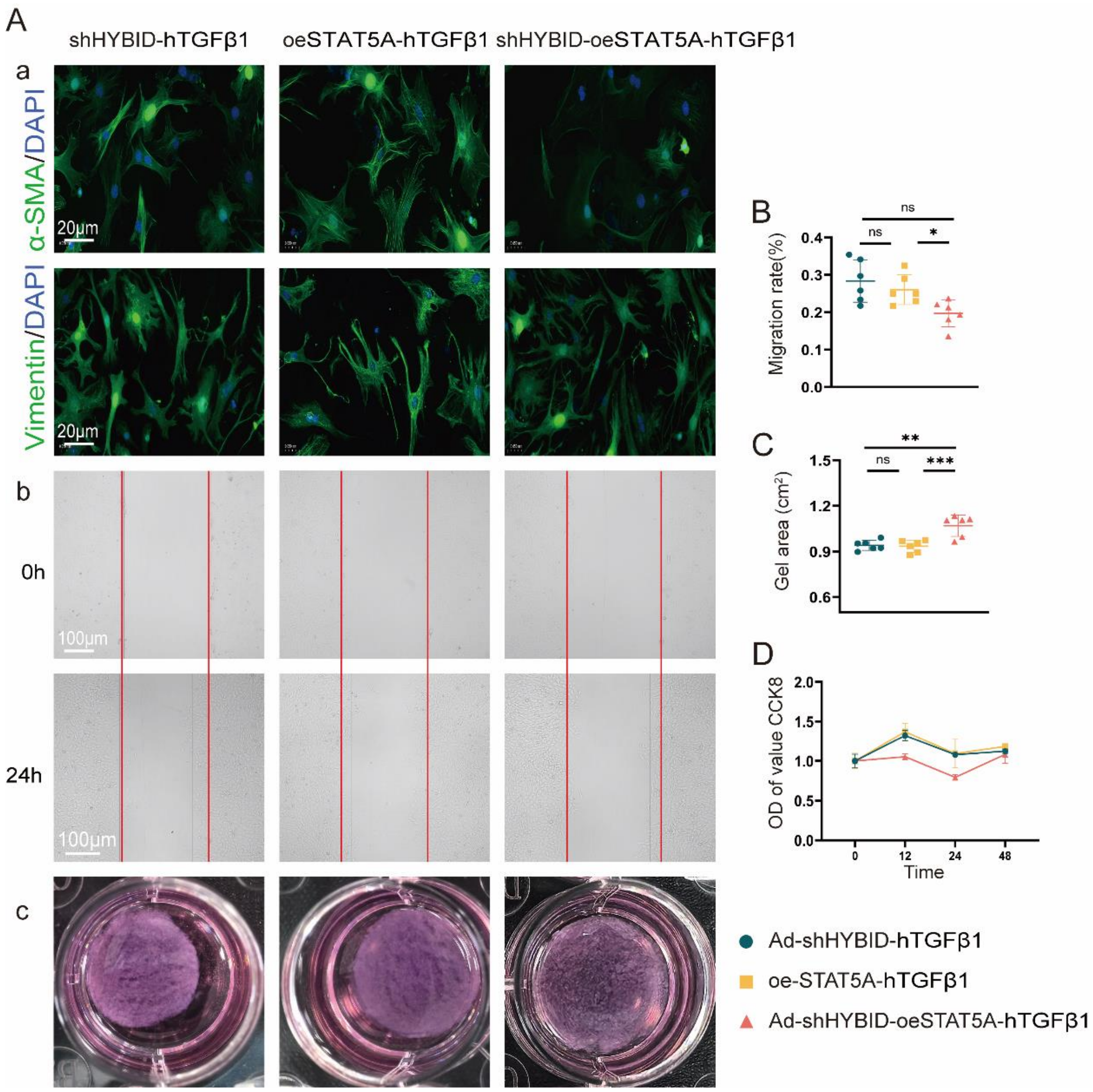

Figure S7 Overexpression of STAT significantly enhances the beneficial effects of shHYBID on cellular functions. A Representative images of immunofluorescence staining(a), scratch the wounds (b) and collagen gel contraction (c) in CFs infected with indicated adenovirus and then treated with PBS or hTGF-β1 for 24 h (n=6). The red lines mark the scratch edges. **B-C** Quantification of migration distances and collagen gel contraction. **D** CCK8 assay to detect fibroblast proliferation. The error bars are the SD. *P<0.05, **P<0.01, **P<0.001, and ****P<0.0001 **B-D** by two-way ANOVA followed by Tukey's multiple comparisons test.

Tables S1 to S3

Table S1. Table of sequences and downstream primers for genes analyzed by qPCR(mouse).

Gene	Forward Primer (5'-3')	Reverse Primer (5'-3')
GAPDH	AGGTCGGTGTGAACGGATTTG	GGGGTCGTTGATGGCAACA
HYBID	GGCCGGTGATGTAGACGAAA	CCATTGGAGCCATGGACTGT
Enpp3	CTCATGCCCTGCACTACAGA	TAGCCTTTGGTTTGCTTGCT
MMP13	GATGACCTGTCTGAGGAAGACC	GCATTTCTCGGAGCCTGTCAAC
Hyou1	TGCGCTTCCAGATCAGTCC	GGAGTAGTTCAGAACCATGCC
Enho	CTCATCGCCATCGTCTGCAAT	GGGACTGGATTCCGAGAGAGA
STAT5A	GTTTGAGTCTCAGTTCAGCGT	CATGGACGATAACGACCACAG

Table S2. Antibody Information.

Target antigen	Vendor or Source	Catalog #	Working concentration
Flag	Proteintech	66008-4-Ig	1: 8000 (WB)<br>3ug (IP)
HA	Proteintech	51064-2-AP	1: 8000 (WB)<br>4ug (IP)
HYBID	Proteintech	21129-1-AP	1: 1000 (WB)<br>1:200 (IF/IHC)
BAX	Proteintech	60267-1-Ig	1: 10000 (WB)
Bcl2	Proteintech	68103-1-PBS	1: 10000 (WB)
STAT5A	Proteintech	13179-1-AP	1: 1000 (WB)<br>1:100 (IF)

MMP13	Proteintech	18165-1-AP	1: 3000 (WB)
α-SMA	Abclonal	A17910	1: 5000 (WB)<br>1:100 (IF)
Collagen I	Abclonal	A16891	1: 1000 (WB)
Collagen III	Abclonal	A0817	1: 3000 (WB)
Vimentin	Abclonal	A19607	1:100 (IF)
GAPDH	Servicebio	GB15004	1: 4000 (WB)
Anti-mouse IgG	Servicebio	GB23301	1:15000 (WB)
Anti-Rabbit IgG	Servicebio	GB23303	1:15000 (WB)

Table S3. Sequence alignment analysis via the JASPAR database

Name	Score	Relative score	Predicted sequence
STAT5A	14.515973	0.971173890547973	gtttctaagaa
JunB	13.7706995	0.9679465403859926	tgatgagtcac
TBP	11.532274	0.9999999888056338	tataaaa
POU3F2	10.128362	0.8593611835977805	atatggtaaaga
Sox2	9.684345	0.8064384373686487	gcattttcatggata
Nkx2-1	8.834246	0.8959717915878463	cacttca
YY1	8.38313	0.9999999883150802	caaaatgg
USF2	6.4629254	0.8639713099805367	gtcatctgaca
